# Socio‐Emotional Skills and Physical Activity in Primary and Secondary Education Students

**DOI:** 10.1111/josh.70152

**Published:** 2026-04-16

**Authors:** Antonio Morcillo‐Martínez, Isabel María Gómez‐Barreto, Álvaro Infantes‐Paniagua, Juan Gregorio Fernández‐Bustos

**Affiliations:** ^1^ Department of Pedagogy Faculty of Education, University of Castilla‐La Mancha Albacete Spain; ^2^ Department of Education Sciences Area of Developmental and Educational Psychology, University of Alcalá Madrid Spain; ^3^ Department of Didactics of Physical Artistic and Music Education, Faculty of Education, University of Castilla‐La Mancha Albacete Spain

**Keywords:** adolescence, childhood, physical activity, social–emotional learning, soft skills

## Abstract

**Background:**

Socio‐emotional skills (SES) are essential for students' wellbeing and academic adjustment, and physical activity (PA) has been shown to contribute positively to emotional, social, and cognitive development. Understanding their relationship within school populations becomes highly relevant for educational and health promotion practices.

**Method:**

A cross‐sectional study was conducted with 874 students aged 10–16 years. SES were assessed according to the OECD framework using the Social and Emotional Skills Survey. PA levels were measured through the physical activity questionnaire for children (PAQ‐C) and adolescents (PAQ‐A), classifying students as active or non‐active.

**Results:**

PA showed positive and significant correlations with all SES domains, with varying intensity across domains. Active students obtained higher SES scores across domains, although small differences by gender and educational level were also observed. Differences were more pronounced at lower educational levels and decreased progressively in higher grades.

**Implications for School Health Policy, Practice, and Equity:**

Findings support integrating PA as a strategic component of social–emotional learning initiatives. Schools can play a key role in promoting students' health and wellbeing by incorporating appropriate PA opportunities that strengthen socio‐emotional learning (SEL) and contribute to a positive school climate.

**Conclusions:**

PA is consistently associated with more favorable socio‐emotional profiles. Promoting structured and meaningful PA in school settings may support students' holistic development and enhance their overall wellbeing.

## 1 Introduction

In an educational overview increasingly focused on students' holistic wellbeing, growing attention has been directed toward those skills that, in addition to supporting personal, social, and academic development, are crucial for adaptive functioning, individual success, and adequate social participation [1, 2, 3, 4]. Socio‐emotional skills (SES) encompass a broad set of functional capabilities that enable individuals to recognize, understand, and regulate emotions, thoughts, and behaviors; interact effectively; establish positive relationships; make responsible decisions; and pursue personal goals [5, 6].

This way of referring to SES acts as an umbrella concept that reflects the breadth and evolving nature of the construct within educational research [7, 8]. Different terms are used in the literature to designate this conceptual space, such as *non‐cognitive skills*, *21st‐century skills*, *transferable skills*, or *soft skills*, among others. Although all these terms share the idea of independence from cognitive skills, several authors have pointed out that there is considerable variability in the number and nature of the skills included in the different models and frameworks, without a conceptual agreement having been reached [3, 4, 9]. This conceptual diversity has produced multiple models that differ in terminology and in the specific skills included, underscoring the complexity and multidimensionality of the field [10, 11] and presenting challenges for valid assessment [12].

Within the scope of this study, the model proposed by the organization for economic cooperation and development (OECD [13]) is particularly relevant, as it offers a coherent theoretical and operational framework for assessing SES in educational contexts [14]. This model groups SES into five general domains—task performance, emotional regulation, collaboration, open‐mindedness, and engaging with others—each comprising three clearly defined skills. The model has been applied in international studies such as the survey on social and emotional skills (SSES) [15, 16] and provides a robust benchmark for systematic SES assessment across educational levels and cultural contexts [17, 18].

### 1.1 Socio‐Emotional Skills in the School Context

Extensive evidence shows that strengthening SES positively influences emotional wellbeing, mental health, social adjustment, employability, and life‐course outcomes [19, 20, 21] and is strongly associated with academic performance [22, 23]. This body of evidence has reinforced the recognition of SES as a fundamental aspect of education policies at the international level, with implications for the organization of educational systems, the definition of curricular frameworks, and the planning of different educational levels in various countries [24].

The development of SES does not follow homogeneous trajectories and is shaped by biological, psycho‐evolutionary, contextual, and sociocultural factors [25, 26]. Among these, age and gender stand out for their consistent influence [16, 27]. Studies indicate that SES varies across developmental stages, with younger students generally exhibiting higher levels of these skills [28, 29]. Gender differences have also been documented: girls typically score higher in socio‐emotional dimensions related to social interaction, whereas boys tend to score higher in skills linked to emotional regulation [30, 31].

In school settings, the promotion of SES has gained priority due to growing concerns about students' mental health and social disconnection [32]. Socio‐emotional learning (SEL) has emerged as a protective strategy that mitigates difficulties related to psychological wellbeing, supports healthy development [33, 34], and has been consistently linked to a lower incidence of emotional, social, and behavioral difficulties, highlighting its preventive value for students' overall wellbeing [35]. Hence, the effectiveness of SEL programs has been widely supported, with numerous reviews and meta‐analyses endorsing their positive impact on improving behavioral problems, reducing symptoms of anxiety and depression, as well as promoting academic performance and school adjustment [36, 37]. These benefits have been replicated across cultural contexts, reinforcing the need for systematic integration of SEL into educational practice [38].

### 1.2 Physical Activity and Socio‐Emotional Skills

Alongside SES research, physical activity (PA), understood as bodily movement that involves energy expenditure and is typically performed through play, sports, exercise, or daily movement behaviors [39], has been widely recognized for its benefits in childhood and adolescence [40].

From a theoretical perspective, the relationship between PA and SES can be interpreted through established frameworks in educational and developmental psychology. Self‐Determination Theory proposes that learning environments supporting autonomy, competence, and relatedness foster motivation and positive youth development [41]. PA contexts, particularly those involving cooperation, challenge, and goal‐oriented tasks, may satisfy these basic psychological needs and thus contribute to the development of SES [42]. Additionally, Social Cognitive Theory emphasizes the role of observational learning, social interaction, and self‐efficacy beliefs in the development of behavior and personal capabilities. Within PA contexts, students interact with peers, experience mastery, and develop self‐efficacy beliefs, processes that are closely linked to the development of emotional regulation, social interaction skills, and adaptive psychosocial functioning [43].

A substantial body of work shows that regular PA yields not only well‐established physiological benefits but also significant emotional, social, and cognitive gains in children and adolescents [44]. PA acts as a protective factor against mental health problems [45, 46], supports emotional regulation, improves self‐esteem, enhances resilience and social skills, and promotes adaptive psychosocial functioning [34, 47, 48, 49]. Additionally, when incorporated systematically into educational contexts, PA has also been associated with improvements in cognitive performance [50].

Consistent with these theoretical perspectives, recent empirical research has reported positive associations between participation in PA and adolescents' SES [51]. Similar findings have also been observed in primary school populations, where higher levels of physical exercise have been associated with more favorable SES profiles [34].

From this point of view, the convergence between SES and PA takes on special relevance [52], as both contribute in a complementary way to the overall wellbeing of students, offering opportunities to implement integrative educational approaches that favor their development from a holistic perspective [53, 54]. Likewise, the literature suggests that physical experiences designed for educational purposes can constitute privileged contexts for social–emotional development, as they promote meaningful interactions and relational learning [55].

In line with these considerations, the main objective of this study was to analyze the relationship between PA practice and the five SES domains defined in the OECD model among Primary and Secondary Education students. Additionally, it sought to examine potential SES differences by gender and educational cycle as well as the combined influence of these variables. Based on the literature, the following hypotheses were proposed: (a) PA practice will be positively associated with all SES domains; (b) active students will show significantly higher SES levels than their non‐active peers; (c) these differences will be more pronounced in earlier educational stages and decrease as schooling progresses; and (d) although gender differences may appear in specific domains, the overall pattern linking PA and SES will be consistent across genders.

## 2 Method

### 2.1 Participants

A quantitative cross‐sectional study was conducted on a sample of 874 students aged between 10 and 16 (M = 12.65; SD = 1.85), from six educational centers – four public and two subsidized– selected through purposive convenience sampling, seeking a balanced representation by gender (51% girls) and educational level. The students ranged from the 5th year of primary education (PE) to 4th year of compulsory secondary education (CSE), which allowed them to be grouped into three educational blocks for subsequent analysis: third cycle of PE (5th and 6th years of PE), first cycle of CSE (1st and 2nd years) and second cycle of CSE (3rd and 4th years). Considering the sample size, a post hoc statistical power analysis conducted using G*Power indicated that the study had sufficient statistical power (1–*β* > 0.80) to detect small effect sizes (*r*≈0.10) at a significance level of *α* = 0.05 [56]. The detailed distribution of the sample by cycle and gender is presented in Table 1.

**TABLE 1:** Distribution of the sample by educational cycle and gender.

Educational cycle	Total (%) M ± SD	Boys (%) M ± SD	Girls (%) M ± SD
3rd cycle of PE	288 (33%) 10.61 ± 0.70	148 (51.4%) 10.54 ± 0.63	140 (48.6%) 10.68 ± 0.76
1st cycle of CSE	331 (37.9%) 12.74 ± 0.77	164 (49.5%) 12.62 ± 0.78	167 (50.5%) 12.86 ± 0.74
2nd cycle of CSE	255 (29.2%) 14.86 ± 0.88	116 (45.5%) 14.96 ± 0.98	139 (54.5%) 14.78 ± 0.78

Abbreviations: CSE, compulsory secondary education; M, mean age (years); PE, primary education; SD, standard deviation.

The educational centers, located in the city of Albacete (Spain), mainly served students from families with middle socioeconomic status and had similar organizational and resource characteristics, with no notable prevalence of students in vulnerable situations.

### 2.2 Instruments

Socio‐emotional skills: the Spanish version of the SSES of the OECD [17] was applied, used internationally to assess SES in two age cohorts (10 and 15 years). In this study, it was applied to all educational levels included in the sample, maintaining the same set of items. The questionnaire, based on the OECD's theoretical model, assesses five SES domains: Task performance, emotional regulation, collaboration, open‐mindedness, and engaging with others. Each domain includes three specific skills, with eight items per skill, for a total of 120 items with Likert scale responses from 1 to 5 (strongly disagree‐strongly agree). The mean values for each domain were calculated from the scores for each skill. The SSES showed adequate psychometric properties in various international contexts. In this study, alpha coefficients ranged from *α* = 0.68 to 0.79, consistent with previously reported ranges (which ranged from 0.68 to 0.92, depending on the version applied [57]).

Physical activity: PA was assessed using the physical activity questionnaire in its versions for children (PAQ‐C) and adolescents (PAQ‐A), both adapted and validated for the Spanish context [58, 59]. The PAQ‐C was applied to students in the 5th–6th years of PE and the PAQ‐A to students in the 1st–4th years of CSE. These self‐reported questionnaires estimate the overall level of PA performed during the last week using a five‐point Likert scale, assessing different moments and contexts of practice; the PAQ‐C includes an additional item on recess time. Both questionnaires have shown adequate psychometric properties in their respective populations, with Cronbach's alpha coefficients of *α* = 0.83 for the PAQ‐C and *α* = 0.74 for the PAQ‐A. In the sample of this study, the reliability indices obtained were *α* = 0.75 (PAQ‐C) and *α* = 0.78 (PAQ‐A), which supports their validity as assessment instruments in their respective age groups. The students were classified as active or non‐active according to the cut‐off points proposed by Benítez‐Porres et al. [60], with those scoring above 2.73 on the PAQ‐C and 2.75 on the PAQ‐A being considered active.

### 2.3 Procedure

The study received the approval of the Social Research Ethics Committee of the University of Castilla‐La Mancha (reference: 723208‐Q4Z4). The management of the educational centers was contacted and informed of the objectives of the research. In those centers that agreed to participate, an informed consent procedure was arranged for the students' legal representatives, ensuring that participation was voluntary, anonymous, and confidential, and respecting the ethical standards established in international research guidelines. Data were only collected from students who had provided duly signed consent. Data collection was carried out physically during school hours, in a single session per group. The questionnaires were completed as self‐reports, at a single time point, with the presence of the research team in the classroom to resolve possible doubts and ensure that the items were correctly understood. The conditions of application were consistent across all centers: a quiet environment was ensured, without interruptions, guaranteeing privacy in the responses. The time taken to complete the questionnaires ranged from 30 to 45 min maximum.

### 2.4 Data Analysis

Statistical analyses were performed using IBM SPSS software (v. 29). Prior to applying inferential tests, the assumptions of normality were verified using the Kolmogorov–Smirnov test and the homogeneity of variances using Levene's test, which allowed parametric procedures to be chosen. First, correlations were calculated to examine associations between PA practice and SES domains. Subsequently, the students were classified into two groups (active and non‐active) following the criteria mentioned above, and based on this classification, multivariate analyses of variance (MANOVA) were carried out to evaluate the effects of PA levels (active vs. non‐active), gender, and educational cycle on the five SES domains. Finally, comparisons were made using Student's *t*‐test for independent samples to analyze the differences between active and non‐active students within each educational cycle. Effect sizes were reported using partial eta squared (*η* ^2^_*p*_) for MANOVA and Cohen's d for *t*‐tests, and a significance level of *p* < 0.05 was adopted. Additionally, multiple and independent regression analyses were conducted to explore the association between SES domains and PA levels while controlling for gender and educational cycle.

## 3 Results

The results, presented in Table 2, indicate positive and statistically significant correlations between PA and all socio‐emotional domains assessed. These associations were particularly consistent in the domains of *Engaging with others* (*r* = 0.44, *p* < 0.01) and *Emotional Regulation* (*r* = 0.31, *p* < 0.01). Overall, the findings suggest that PA practice is linked to more favorable socio‐emotional development, reflected in significant associations with the different dimensions of SES.

**TABLE 2:** Pearson correlations between physical activity and social–emotional domains.

	PA	TP	OM	ER	EO
TP	0.22**	—			
OM	0.24**	0.48**	—		
ER	0.31**	0.48**	0.22**	—	
EO	0.44**	0.22*	0.37**	0.41**	—
CO	0.28**	0.54**	0.56**	0.39**	0.38**

Abbreviations: CO, collaboration; EO, engaging with others; ER, emotional regulation; OM, open‐mindedness; PA, physical activity; TP, task performance.

* *p* < 0.05.

** *p* < 0.01.

In order to jointly examine the combined effects of the PA group (active vs. non‐active), gender, and educational level on the five SES domains, a multivariate analysis of variance (MANOVA) was performed. The results, presented in Table 3, show that the PA group is a determining factor in all socio‐emotional domains (*p* < 0.001), with the largest effect observed in *Engaging with others* (*η* ^2^_*p*_ = 0.108). These results suggest that PA practice is consistently associated with several key components of social–emotional development.

**TABLE 3:** Results of MANOVA analysis: Effects of physical activity, gender, and educational level on socio‐emotional skill domains.

SES domain	PA (active/non‐active)	Gender	Educational level	PA × gender × level interaction
TP	*F*(1, 842) = 17.69, *p* < 0.001, *η* ^2^_*p*_ = 0.021	*F*(1, 842) = 6.90, *p* = 0.009, *η* ^2^_*p*_ = 0.008	*F*(2, 842) = 17.70, *p* < 0.001, *η* ^2^_*p*_ = 0.04	*F*(2, 842) = 0.17, *p* = 0.847, *η* ^2^_*p*_ < 0.001
OM	*F*(1, 842) = 25.23, *p* < 0.001, *η* ^2^_*p*_ = 0.029	*F*(1, 842) = 12.35, *p* < 0.001, *η* ^2^_*p*_ = 0.014	*F*(2, 842) = 8.39, *p* < 0.001, *η* ^2^_*p*_ = 0.02	*F*(2, 842) = 0.10, *p* = 0.905, *η* ^2^_*p*_ < 0.001
ER	*F*(1, 842) = 32.91, *p* < 0.001, *η* ^2^_*p*_ = 0.038	*F*(1, 842) = 54.20, *p* < 0.001, *η* ^2^_*p*_ = 0.06	*F*(2, 842) = 11.69, *p* < 0.001, *η* ^2^_*p*_ = 0.027	*F*(2, 842) = 0.39, *p* = 0.676, *η* ^2^_*p*_ = 0.001
EO	*F*(1, 842) = 102.26, *p* < 0.001, *η* ^2^_*p*_ = 0.108	*F*(1, 842) = 13.20, *p* < 0.001, *η* ^2^_*p*_ = 0.015	*F*(2, 842) = 0.84, *p* = 0.432, *η* ^2^_*p*_ = 0.002	*F*(2, 842) = 1.07, *p* = 0.344, *η* ^2^_*p*_ = 0.003
CO	*F*(1, 842) = 39.00, *p* < 0.001, *η* ^2^_*p*_ = 0.044	*F*(1, 842) = 5.57, *p* = 0.019, *η* ^2^_*p*_ = 0.007	*F*(2, 842) = 20.49, *p* < 0.001, *η* ^2^_*p*_ = 0.046	*F*(2, 842) = 0.39, *p* = 0.676, *η* ^2^_*p*_ = 0.001

Abbreviations: CO, collaboration; EO, engaging with others; ER, emotional regulation; OM, open‐mindedness; PA, physical activity; SES, socio‐emotional skills; TP, task performance.

Focusing on gender, significant main effects were observed in all domains, including *Task Performance* (*p* = 0.009), *Open‐mindedness* (*p* < 0.001), *Emotional Regulation* (*p* < 0.001; *η* ^2^_*p*_ = 0.06), *Engaging with others* (*p* < 0.001), and *Collaboration* (*p* = 0.019). Regarding educational level, main effects were observed in *Task Performance* (*p* < 0.001), *Open‐mindedness* (*p* < 0.001), *Emotional Regulation* (*p* < 0.001), and *Collaboration* (*p* < 0.001), indicating variations between cycles in these domains. In contrast, no significant differences were found in *Engaging with others* (*p* = 0.432). Finally, no interaction effects between PA, gender, and educational level were identified (*p* ≥ 0.05), suggesting that these factors act independently in the development of SES.

To further explore the relationship between SES and PA, additional regression analyses were conducted controlling for gender and educational cycle. First, a multiple regression analysis was performed including the five SES domains as predictors of PA. The overall model was statistically significant and explained a moderate proportion of variance in PA levels (*R* ^2^ = 0.298, *p* < 0.001; see Table S1). Additionally, independent regression analyses were conducted to examine the contribution of each SES domain to PA levels. The results indicated that the Engaging with Others domain showed the strongest association with PA. Detailed statistical outputs of these regression analyses are provided in Supporting Information (see Tables S2–S6).

In order to examine in greater detail the differences in the socio‐emotional domains according to the level of PA, comparisons were made between students classified as active and non‐active in each educational cycle. The results in Table 4 show significant differences in most of the socio‐emotional domains in favor of the active group, especially in the case of PE and the first cycle of CSE.

**TABLE 4:** Comparison of socio‐emotional domains between active and inactive students by educational level.

Educational level	SES domain	Group	*n*	M ± SD	Student's *t*	*p*	*d* (95% CI)
Third cycle of PE (5th and 6th PE)	TP	Active	187	30.73 ± 4.09	2.01	0.02	0.25 (0.50, 0.01)
		Non‐active	95	29.66 ± 4.55			
	OM	Active	187	31.55 ± 3.80	3.30	< 0.001	0.42 (0.67, 0.17)
		Non‐active	95	29.88 ± 4.36			
	ER	Active	188	28.51 ± 4.28	4.61	< 0.001	0.58 (0.83, 0.33)
		Non‐active	96	25.93 ± 5.76			
	EO	Active	187	28.66 ± 3.65	7.52	< 0.001	0.94 (1.21, 0.69)
		Non‐active	95	25.03 ± 4.18			
	CO	Active	186	32.12 ± 3.30	4.04	< 0.001	0.51 (0.76, 0.26)
		Non‐active	95	30.33 ± 3.89			
First cycle of CSE (1st and 2nd CSE)	TP	Active	175	29.09 ± 3.87	4.06	< 0.001	0.45 (0.67, 0.23)
		Non‐active	151	27.17 ± 4.68			
	OM	Active	175	29.77 ± 4.23	1.95	< 0.001	0.22 (0.44, 0.002)
		Non‐active	151	28.85 ± 4.32			
	ER	Active	174	26.93 ± 4.58	6.23	< 0.001	0.69 (0.92, 0.47)
		Non‐active	151	23.74 ± 4.62			
	EO	Active	175	27.87 ± 4.03	6.22	< 0.001	0.69 (0.92, 0.47)
		Non‐active	151	24.96 ± 4.42			
	CO	Active	174	30.19 ± 4.04	3.77	< 0.001	0.42 (0.64, 0.20)
		Non‐active	151	28.55 ± 3.83			
Second cycle of CSE (3rd and 4th CSE)	TP	Active	91	28.59 ± 4.04	0.58	0.28	0.08 (0.33, −0.18)
		Non‐active	162	28.28 ± 4.09			
	OM	Active	92	30.25 ± 3.65	2.24	0.01	0.29 (0.55, 0.03)
		Non‐active	162	29.10 ± 4.08			
	ER	Active	92	27.10 ± 5.01	2.16	0.005	0.34 (0.60, 0.08)
		Non‐active	162	25.43 ± 3.94			
	EO	Active	92	28.00 ± 3.67	5.91	< 0.001	0.77 (1.04, 0.51)
		Non‐active	162	25.03 ± 3.94			
	CO	Active	92	29.97 ± 3.28	2.51	0.006	0.33 (0.58, 0.07)
		Non‐active	162	28.91 ± 3.24			

Abbreviations: CI, confidence interval; CO, collaboration; CSE, compulsory secondary education; *d*, Cohen's *d*; EO, engaging with others; ER, emotional regulation; M, mean; OM, open‐mindedness; *p*, *p*‐value; PE, primary education; SD, standard deviation; TP, task performance.

In PE, active students scored significantly higher in all domains, with particularly relevant effect sizes in *Engaging with others* (*d* = 0.94) and *Emotional Regulation* (*d* = 0.58), followed by *Collaboration* (*d* = 0.51), *Open‐mindedness* (*d* = 0.42), and *Task Performance* (*d* = 0.25). In the first cycle of CSE, this pattern was maintained, with significant differences observed in the five domains. The most pronounced effects again corresponded to *Engaging with others* (*d* = 0.69) and *Emotional Regulation* (*d* = 0.61), while the differences were moderate in *Collaboration* (*d* = 0.42) and *Task Performance* (*d* = 0.45), and smaller in *Open‐mindedness* (*d* = 0.22). In the second cycle of CSE, the differences in favor of active students were more attenuated in several domains, with significant differences observed in *Engaging with others* (*d* = 0.77), *Collaboration* (*d* = 0.33), *Emotional Regulation* (*d* = 0.35), and *Open‐mindedness* (*d* = 0.29), as well as the absence of significant differences in the case of *performance in tasks* (*p* = 0.28).

These findings, in line with MANOVA results, indicate that significant differences in favor of the active group are consistently manifested in both genders, suggesting that PA is associated with broader socio‐emotional development regardless of gender and differences between educational levels.

## 4 Discussion

The aim of this study was to analyze the relationship between levels of PA practice and the development of SES in Primary and Secondary Education students. The results support this association, showing that higher levels of PA are linked to a more favorable socio‐emotional profile in the school population. Correlational analyses revealed positive and statistically significant associations between PA and the five SES domains defined in the OECD model, especially in Engaging with others and Emotional Regulation. Complementary regression analyses also indicated that the Engaging with others domain showed the strongest association with PA levels. This pattern is consistent with previous research demonstrating the contribution of PA to social skills, cooperation, self‐esteem, and emotional coping [47, 48, 49]. Overall, the findings suggest that PA promotes experiences of positive interaction and affective regulation that support students' socio‐emotional development [37].

The MANOVA results indicated that PA is a determining factor across all SES domains, with significant effects on Task Performance, Open‐mindedness, Emotional Regulation, Engaging with others, and Collaboration. Notably, the largest effect was observed in Engaging with others (*η* ^2^_*p*_ = 0.108), suggesting that PA may be relevant for promoting interpersonal interaction and social engagement among students. These results coincide with studies highlighting the influence of PA on self‐regulation, resilience, and social competencies [34, 44, 61], reinforcing the idea that more active students exhibit better socio‐emotional adjustment than their less active peers. Regarding educational level, significant differences emerged in Task Performance, Open‐mindedness, Emotional Regulation, and Collaboration, indicating variations between cycles in these domains, whereas Engaging with others did not show significant differences. These findings suggest that certain SES may evolve alongside developmental and contextual changes associated with schooling [26, 28]. Gender also showed significant main effects across all domains, although the effect sizes were generally small, indicating modest differences between boys and girls in their socio‐emotional profiles.

Comparisons between active and non‐active students across educational levels confirmed that differences in favor of the active group were consistently observed in most domains, particularly in the last cycle of PE and the first cycle of CSE. These differences were less pronounced in the second cycle of CSE, suggesting a decline in the impact of PA on socio‐emotional development with increasing age. This pattern could be explained by developmental and motivational processes typical of adolescence, such as the pursuit of autonomy, changing social interests, or reduced engagement in structured PA [25]. Although descriptive gender differences were observed in some domains, the relatively small effect sizes indicate that the socio‐emotional benefits associated with PA appear broadly comparable between boys and girls, in line with studies documenting generalizable effects of PA on the wellbeing of children and adolescents [16, 47, 48].

### 4.1 Implications for School Health Policy, Practice, and Equity

From an applied perspective, educational institutions play an essential role as promoters of SEL [15, 16]. Considering PA as a pedagogical resource for SEL, beyond its recreational or health value, implies leveraging its potential to foster self‐regulation, cooperation, and social interaction [36, 38]. Integrating PA activities designed with socio‐emotional objectives can improve school climate and simultaneously support students' wellbeing and academic learning [33, 35].

The present findings also have implications for school health policy and educational equity. Since higher levels of PA were associated with more favorable SES profiles across domains, schools may represent a key setting for ensuring that all students, regardless of gender or educational stage, have access to structured and meaningful opportunities for movement and participation [34, 53]. From an equity perspective, promoting PA within the school context may be especially relevant because it offers a universal and potentially accessible pathway for supporting socio‐emotional development in diverse student populations [7, 21]. Thus, PA should be considered not only a health‐promoting behavior but also a strategic educational resource that can contribute to more inclusive and supportive school environments [54].

### 4.2 Limitations

Despite the relevance of the results, certain limitations must be acknowledged. First, the cross‐sectional design prevents establishing causal relationships, so future research should incorporate longitudinal or quasi‐experimental designs that allow the directionality and intensity of the relationship between PA and SES to be clarified [32]. Second, the reliance on self‐reported measures may introduce memory or social desirability biases; thus, combining these instruments with objective or observational measures would enhance validity. Additionally, although the sample was large and diverse in age and gender, it was obtained through convenience sampling, limiting generalizability. Replication in broader and more heterogeneous contexts is therefore recommended. Moreover, the study does not differentiate the type of PA or the pedagogical approaches used in organized contexts, which constrains interpretation. It is likely that the educational impact of PA on SES is strengthened when activities are intentionally planned and implemented, given that many socio‐emotional behaviors require explicit teaching to develop [55].

In this regard, future research should explore the mediating mechanisms through which PA influences the different socio‐emotional domains and examine the moderating role of personal and contextual variables (e.g., self‐concept, school climate). It would also be valuable to analyze differences based on types and contexts of PA practice, since various modalities and methodologies may influence SES domains differently. For example, cooperative or team‐based activities may enhance collaboration and interpersonal skills, whereas individual activities may contribute more to task performance and emotional regulation [50, 61]. Additionally, the relationship between PA and SES may be bidirectional: regular PA could strengthen SES, while higher SES levels may, in turn, encourage engagement in active habits, as suggested by models of reciprocal interaction between socio‐emotional wellbeing and healthy behavior [19, 21]. Exploring this reciprocity would improve understanding of the mechanisms underpinning the relationship and guide more effective educational strategies, aligned with the priorities of the OECD [15, 16] and UNESCO [6] on holistic education and student wellbeing.

Overall, the findings of this study provide robust empirical evidence of the positive relationship between regular PA and socio‐emotional development in school populations, showing that more active students exhibit more favorable SES profiles. These results align with the increasing international emphasis on promoting student wellbeing and strengthening SES for personal, social, and academic adjustment [1, 4, 24]. They also reinforce the recommendations of organizations advocating for SEL as a key component of quality education [6, 38] and recognize PA as a determinant of healthy development due to its psychological, emotional, and relational benefits [34, 44, 45]. From an applied standpoint, these findings highlight the need to conceive PA not merely as recreation but as a strategic educational tool capable of promoting policies and practices aimed at students' holistic development.

## Conflicts of Interest

The authors declare no conflicts of interest.

## Supporting information


**Table S1:** Multiple regression analysis predicting PA from SES domains controlling for gender and educational level.
**Table S2:** Multiple regression model predicting PA from Task Performance domain controlling for gender and educational level.
**Table S3:** Multiple regression model predicting PA from Open‐mindedness domain controlling for gender and educational level.
**Table S4:** Multiple regression model predicting PA from Emotional Regulation domain controlling for gender and educational level.
**Table S5:** Multiple regression model predicting PA from Engaging with Others domain controlling for gender and educational level.
**Table S6:** Multiple regression model predicting PA from Collaboration domain controlling for gender and educational level.

## Data Availability

The data that support the findings of this study are available on request from the corresponding author. The data are not publicly available due to privacy or ethical restrictions.
