# Supplementary material for: Socio‐Emotional Skills and Physical Activity in Primary and Secondary Education Students
Source: J Sch Health. 2026 Apr 16;96:e70152. doi: 10.1111/josh.70152 (PMC13084471; doi:10.1111/josh.70152)
Supplement: Supplementary file 1 — Table S1: Multiple regression analysis predicting PA from SES domains controlling for gender and educational level. Table S2: Multiple regression model predicting PA from Task Performance domain controlling for gender and educational level. Table S3: Multiple regression model predicting PA from Open‐mindedness domain controlling for gender and educational level. Table S4: Multiple regression model predicting PA from Emotional Regulation domain controlling for gender and educational level. Table S5: Multiple regression model predicting PA from Engaging with Others domain controlling for gender and educational level. Table S6: Multiple regression model predicting PA from Collaboration domain controlling for gender and educational level. [file JOSH-96-0-s001.docx]

**SUPPLEMENTARY MATERIALS**

**Supplementary Table S1. *Multiple regression analysis predicting PA from SES domains controlling for gender and educational level.***

| **Predictor** | **B** | **SE** | **β** | **t** | **p** |
| --- | --- | --- | --- | --- | --- |
| Gender | -.242 | .042 | -.182 | -5.771 | < .001 |
| Educational level | -.188 | .025 | -.222 | -7.464 | < .001 |
| Task Performance | .009 | .006 | .062 | 1.583 | .114 |
| Open-mindedness | .008 | .006 | .047 | 1.269 | .205 |
| Emotional Regulation | .006 | .005 | .043 | 1.133 | .257 |
| Engaging with others | .048 | .005 | .311 | 9.023 | < .001 |
| Collaboration | .005 | .007 | .031 | .785 | .433 |
| **Model statistics:** R² = .298; ΔR² = .144 | | | | | |
| Note: B = unstandardized regression coefficient; SE = standard error; β = standardized regression coefficient; t = t statistic; p = significance level. The dependent variable was physical activity (PA). | | | | | |

**Supplementary Table S2. *Multiple regression model predicting PA from Task Performance domain controlling for gender and educational level.***

| **Predictor** | **B** | **SE** | **β** | **t** | **p** |
| --- | --- | --- | --- | --- | --- |
| Constant | 2.919 | .166 | - | 45.82 | < .001 |
| Gender | -.348 | .041 | -.262 | -8.509 | < .001 |
| Educational level | -.217 | .026 | -.258 | -8.229 | < .001 |
| Task Performance | .029 | .005 | .187 | 5.972 | < .001 |
| **Model statistics:** R² = .189; ΔR² = .034 | | | | | |

*Note.* B = unstandardized regression coefficient; SE = standard error; β = standardized regression coefficient; t = t statistic; p = significance level; R² = proportion of explained variance; ΔR² = increase in explained variance after adding the SES domain to the model controlling for gender and educational cycle. Dependent variable: physical activity (PA).

**Supplementary Table S3. *Multiple regression model predicting PA from Open-mindedness domain controlling for gender and educational level.***

| **Predictor** | **B** | **SE** | **β** | **t** | **p** |
| --- | --- | --- | --- | --- | --- |
| Constant | 2.693 | .170 | - | 15.87 | < .001 |
| Gender | -.357 | .041 | -.268 | -8.744 | < .001 |
| Educational level | -.216 | .026 | -.256 | -8.293 | < .001 |
| Open-mindedness | .036 | .005 | .224 | 7.237 | < .001 |
| **Model statistics:** R² = .201; ΔR² = .049 | | | | | |
| *Note.* B = unstandardized regression coefficient; SE = standard error; β = standardized regression coefficient; t = t statistic; p = significance level; R² = proportion of explained variance; ΔR² = increase in explained variance after adding the SES domain to the model controlling for gender and educational cycle. Dependent variable: physical activity (PA). | | | | | |

**Supplementary Table S4. *Multiple regression model predicting PA from Emotional Regulation domain controlling for gender and educational level.***

| **Predictor** | **B** | **SE** | **β** | **t** | **p** |
| --- | --- | --- | --- | --- | --- |
| Constant | 2.802 | .162 | - | 17.31 | < .001 |
| Gender | -.246 | .043 | -.185 | -5.780 | < .001 |
| Educational level | -.222 | .026 | -.263 | -8.514 | < .001 |
| Emotional Regulation | .030 | .004 | .223 | 6.936 | < .001 |
| **Model statistics:** R² = .197; ΔR² = .045 | | | | | |
| *Note.* B = unstandardized regression coefficient; SE = standard error; β = standardized regression coefficient; t = t statistic; p = significance level; R² = proportion of explained variance; ΔR² = increase in explained variance after adding the SES domain to the model controlling for gender and educational cycle. Dependent variable: physical activity (PA). | | | | | |

**Supplementary Table S5. *Multiple regression model predicting PA from Engaging with Others domain controlling for gender and educational level.***

| **Predictor** | **B** | **SE** | **β** | **t** | **p** |
| --- | --- | --- | --- | --- | --- |
| Constant | 1.997 | .161 | - | 12.37 | < .001 |
| Gender | -.221 | .040 | -.166 | -5.584 | < .001 |
| Educational level | -.208 | .025 | -.246 | -8.425 | < .001 |
| Engaging with Others | .058 | .005 | .373 | 12.479 | < .001 |
| **Model statistics:** R² = .280; ΔR² = .131 | | | | | |
| *Note.* B = unstandardized regression coefficient; SE = standard error; β = standardized regression coefficient; t = t statistic; p = significance level; R² = proportion of explained variance; ΔR² = increase in explained variance after adding the SES domain to the model controlling for gender and educational cycle. Dependent variable: physical activity (PA). | | | | | |

**Supplementary Table S6. *Multiple regression model predicting PA from Collaboration domain controlling for gender and educational level.***

| **Predictor** | **B** | **SE** | **β** | **t** | **p** |
| --- | --- | --- | --- | --- | --- |
| Constant | 2.502 | .193 | - | 12.94 | < .001 |
| Gender | -.339 | .041 | -.255 | -8.323 | < .001 |
| Educational level | -.198 | .027 | -.234 | -7.440 | < .001 |
| Collaboration | .040 | .005 | .227 | 7.232 | < .001 |
| **Model statistics:** R² = .199; ΔR² = .049 | | | | | |
| *Note.* B = unstandardized regression coefficient; SE = standard error; β = standardized regression coefficient; t = t statistic; p = significance level; R² = proportion of explained variance; ΔR² = increase in explained variance after adding the SES domain to the model controlling for gender and educational cycle. Dependent variable: physical activity (PA). | | | | | |
